# Circulating Biomarkers of Thyroid Cancer: An Appraisal

**DOI:** 10.3390/jcm14051582

**Published:** 2025-02-26

**Authors:** Marta Codrich, Alessia Biasotto, Federica D’Aurizio

**Affiliations:** 1 Department of Medicine (DMED), University of Udine, 33100 Udine, Italy; marta.codrich@uniud.it (M.C.); alessia.biasotto@asufc.sanita.fvg.it (A.B.); 2 Institute of Clinical Pathology, Academic Hospital “Santa Maria della Misericordia”, Azienda Sanitaria Universitaria Friuli Centrale, 33100 Udine, Italy

**Keywords:** circulating biomarkers, thyroid cancer, circulating tumor cells, circulating tumor nucleic acids, circulating tumor DNAs, circulating tumor RNAs

## Abstract

Thyroid cancer is the most prevalent endocrine cancer. The prognosis depends on the type and stage at diagnosis. Thyroid cancer treatments involve surgery, possibly followed by additional therapeutic options such as hormone therapy, radiation therapy, targeted therapy and chemotherapy. Besides the well-known thyroid tumor biomarkers, new circulating biomarkers are now emerging. Advances in genomic, transcriptomic and proteomic technologies have allowed the development of novel tumor biomarkers. This review explores the current literature data to critically analyze the benefits and limitations of routinely measured circulating biomarkers for the diagnosis and monitoring of thyroid cancer. The review also sheds light on new circulating biomarkers, focusing on the challenges of their use in the clinical management of thyroid cancer, underlining the need for the identification of a new generation of circulating biomarkers.

## 1. Introduction

The first use of blood biomarkers to detect and manage malignant thyroid tumors was dated almost 50 years ago [1,2]. Over the years, several circulating biomarkers have been identified and routinely used in clinical practice for the management of thyroid cancer, especially after primary treatment, to identify timely residual disease and recurrent or distant metastasis. Circulating biomarkers of thyroid cancer mainly include peptides and proteins, expressed on the cell surface or secreted into the bloodstream [3]. Recent approaches, due to the advent of new technologies such as genomics, transcriptomics and proteomics, have greatly expanded the range of blood biomarkers, considering also circulating tumor cells and circulating tumor nucleic acids, including cell-free and cell-derived exosome DNA and RNA, released from cancer cells into the bloodstream [3,4,5]. These markers mirror the tumor-specific characteristics, monitoring the development of the cancer disease [4,5]. This review focuses on blood biomarkers used in the clinical management of patients with thyroid cancer. In detail, we aim to both describe the advantages and limitations of circulating biomarkers commonly used in routine and highlight the challenges of new potential circulating biomarkers arising from technological developments and recent discoveries in the field of cancer. For this manuscript, the literature review was performed using the bibliographic database Pubmed. The search keywords were “thyroid cancer” and “circulating biomarkers” with no time restrictions, focusing on the latest reviews and research articles as well as on included studies of the chosen publications.

## 2. Thyroid Cancer

Thyroid cancer is the most common endocrine neoplasm [6] and accounts for about 2.2% of all new cancer cases [7]. The risk of developing thyroid cancer is higher in females and increases with age [8]. The classification of thyroid neoplasms is based on histopathology and molecular pathogenesis according to the fifth edition of the World Health Organization (WHO) thyroid cancer categorization released in 2022 [9]. Thyroid cancer mainly includes the tumors arising from both parafollicular cells (C cells) giving rise to the medullary thyroid carcinoma (MTC), belonging to the dispersed neuroendocrine system, and from follicular epithelial cells resulting in papillary thyroid carcinoma (PTC), follicular thyroid carcinoma (FTC), oncocytic thyroid carcinoma (OTC), high-grade non-anaplastic carcinoma or anaplastic thyroid carcinoma (ATC) [9]. The transformation that occurs in thyroid cancer is caused by mutations in genes that encode mainly for molecules involved in cell proliferation and apoptosis, thus triggering aggressiveness, dedifferentiation, and decreased or no response to therapy [10]. The most common genetic modifications occur in BRAF, RAS, TERT, RET, TP53 genes and RET/PTC gene fusion with various distributions in different histotypes of thyroid cancer [10,11]. WHO 2022 classification for thyroid tumors shows a greater interest in characterizing neoplastic lesions from a molecular perspective. Histological and molecular terminology is then used for any specific microscopic morphology related to well-known genetic alterations [9].

PTC and FTC are the most common histological types [12]. PTC, FTC and OTC are collectively referred to as differentiated thyroid carcinoma (DTC) [13] because tumor cells recollect some of the properties of normal thyrocytes, in particular, the ability to respond to stimulation by thyrotropin (TSH) and absorb and store iodine [12]. PTC accounts for about 80–85% of thyroid cancer in adults and 90% in the pediatric population [12,14]. Despite its high prevalence among endocrine neoplasms, DTC has an excellent prognosis with a 5-year survival rate of 98.4% of cases [7], especially if it is detected early and treated promptly [15]. However, regular follow-up is essential to monitor for signs of recurrence [15]. DTC usually does not cause symptoms in the early stages of the disease with normal thyroid function [16] and the clinical presentation of DTC in many cases is a solitary thyroid nodule [12]. Although most thyroid nodules are benign, a thorough examination is necessary to rule out cancer patients [16]. In this respect, imaging plays a crucial role in DTC diagnostics. Undoubtedly, ultrasonography provides detailed information on the characteristics of nodules, such as size, composition and vascularity [17,18] which, considered together and never individually, support the diagnostic accuracy of the malignancy of a thyroid nodule [19]. Ultrasound-guided fine needle aspiration biopsy (FNAB) with subsequent cytological examination of the specimen can be used to determine the benign or malignant nature of nodules [18], and it is mandatory in rapidly growing nodules, especially in younger patients [12]. However, this approach has several challenges including a high rate of inadequate sampling, the presence of a lot of blood that can obscure thyrocytes due to the extensive perfusion of the thyroid, and the frequent difficulty in follicular neoplasms to distinguish benign from malignant forms [20]. Overall, indeterminate or suspected lesions account for 15–25% of cases investigated by the FNAB, with about 30% eventually being malignant [21]. This last finding was obtained in several studies that used different cytological classifications [21], Thy 3 according to British Thyroid Association Guidelines [22], Class 3 by American Association Clinical Endocrinologists/Associazione Medici Endocrinologi and European Thyroid Association guidelines [23], or Categories III–IV of the Bethesda System for Reporting Thyroid Cytopathology [24].

The knowledge of the molecular etiology of thyroid cancer has provided the basis for a better understanding of cytologically indeterminate nodules. The introduction of molecular techniques, including polymerase chain reaction (PCR) and next-generation sequencing (NGS), has allowed the detection of the driver alterations involved in thyroid cancer development directly on FNA samples. Nowadays, there are different NGS-based assays available for clinical practice. In brief, these tests can detect not only the point gene mutations, gene–gene fusions, and insertions/deletions located in the driver genes mainly associated with components of MAPK and PI3K/Akt pathways, but also copy number alterations, differentially expressed genes, and/or miRNAs linked to the various types of thyroid cancers. In detail, ThyroSeq v.3 explores 112 thyroid cancer-related genes; Afirma Gene Sequencing Classifier analyzes RNAs’ expression combining machine learning algorithms; and ThyGeNEXT/ThyraMIR merges mutational panel with miRNAs’ expression. These assays are mostly used to discriminate between benign and malignant thyroid nodules and have allowed a significant reduction in unnecessary thyroid surgeries for suspicious nodules [25,26]. The main treatment for DTC is surgery, which aims to remove the tumor mass and possibly nearby lymph nodes [27]. The type of surgery to be performed depends on the size of the tumor, the presence of metastases, and the histology of the subtype [27]. In Americas, Europe, and much of Australasia, (near-) total thyroidectomy is usually performed in almost all patients [6]. Only for pT1a PTC, the hemithyroidectomy is considered sufficient [28,29]; however, some authors suggest only observation without surgery resection [6]. More recently, a discussion has arisen about the need for total thyroidectomy in non-locally invasive and non-metastatic DTC with a tumor diameter of up to 4 cm [28]; however, this strategy has yet to prove its efficacy [30]. Most current guidelines recommend the post-surgical radioactive iodine (RAI) ablation as a second component of the primary treatment of DTC in most thyroidectomized patients [28]. Specifically, RAI therapy is used to target and eliminate residual cancer cells and any surviving thyroid tissue [28]. External radiation therapy is used for situations where RAI ablation and surgery are not sufficient to manage aggressive or advanced thyroid carcinomas that do not respond to standard treatments [31]. Moreover, the treatment of resistant DTC includes immune checkpoint inhibitors which stimulate the immune system against cancer cells [31,32] and drugs targeting gene abnormalities to limit the proliferation of cancer cells [33,34].

On the other hand, MTC is a rare thyroid carcinoma that accounts for approximately 1.4–5% of all thyroid malignancies [35,36]. In general, MTC is more aggressive than follicular cell-derived carcinoma with lymph node involvement and sometimes with distant metastases at the time of diagnosis [37]. Although lower than the incidence of DTC, the incidence of MTC has increased in the last three decades, from 0.14 to 0.21 per 100,000 people [38], thanks to the introduction of serum calcitonin (CT) as a screening test in multinodular goiter and to the use of high-resolution ultrasound [39,40]. MTC is inherited in 25% of cases, due to mutations in RET proto-oncogene observed in the context of multiple endocrine neoplasia (MEN) syndromes or familial medullary thyroid cancer (FMTC) [37]. Hereditary MTC is often bilateral, multicentric, and associated with C-cell hyperplasia. Patients with inherited MTC may present with systemic manifestations as a result of excessive secretion of hormones from the tumor, which includes CT and its related peptides. Patients may also present with manifestations of MEN syndromes [37]. Sporadic MTC appears as a firm, hard nodule in the mid-upper region of the thyroid lobes where C cells predominate [41]. At presentation, most patients (70%) with sporadic disease have lymph node involvement and approximately 10% of them have distant metastases [37]. Preoperative ultrasonography typically shows non-specific features of malignancy [41], and cytology is diagnostic in only 50% of cases [39]. Adequate imaging should be performed to determine the extent of the disease. Besides CT and MRI, nuclear medicine techniques, such as PET/CT with 18F-FDOPA and 18F-FDG are used in the evaluation of disease extension for pre-operative staging and post-operative follow-up [42]. The initial treatment of MTC is surgical. Total thyroidectomy is recommended due to the high incidence of multifocal and bilateral disease, especially in patients with sporadic MTC [43]. In addition to total thyroidectomy, central lymph node compartment dissection prophylaxis is performed even if no lymph node involvement and no evidence of distant metastases have been detected in pre-operative staging [35]. Conversely, total thyroidectomy with lateral and central compartment dissection is advisable in cases with preoperatively confirmed cervical lymph node involvement [35]. In patients with basal CT levels greater than 200 ng/L with no evidence of distant metastasis, it is recommended to complete the intervention with prophylactic dissection of uninvolved contralateral neck compartments [35].

Finally, an essential part of postoperative treatment for both DTC and MTC is thyroid hormone replacement therapy. As thyroidectomy causes loss of thyroid function, patients should take thyroid hormone supplements to restore euthyroidism and to suppress TSH for cancer control [44].

## 3. Blood Biomarkers in Thyroid Cancer: State of Art

### 3.1. Thyroglobulin

Thyroglobulin (TG) is a glycoprotein of 660 kDa produced by thyroid follicular cells, and it provides the substrate for the synthesis of thyroid hormones, which are T4 and T3. TG is produced by both normal and tumor thyroid cells, thus indicating the presence of thyroid tissue [45]. In DTC, although not useful as a diagnostic marker, TG is considered the most reliable marker for the identification of disease persistence or recurrence after thyroidectomy and adjuvant administration of RAI ablation [28]. Indeed, the serum concentration of TG, usually measured with continued T4 treatment (onT4-TG) should be undetectable upon tumor thyroid tissue removal as indicated by the follow-up algorithms of the American Thyroid Association [28]. In clinical practice, the measurement of serum TG is based on immunometric assays (IMAs) starting from the first radio immunoassay (RIA) and immunoradiometric assay (IRMA) based on radioactive labeled TG, continuing with immunoluminometric assay (ILMA), chemiluminescence enzyme immunoassay (CLEIA) and chemiluminescence assays (CLIA) based on light emission, fluoroimmunoassay (FIA), fluorescence enzyme immunoassay (FEIA) and enzyme-linked fluorescence assay (ELFA) based on fluorescence emission, only to end with electrochemiluminescence assays (ECLIA), based on chemiluminescent reaction after applying a voltage and Time Resolved Amplified Cryptate Emission (TRACE) technology based on energy transferring [46,47]. The implementations of commercial TG assays with high analytical sensitivity (hsTG) have allowed an increase in their performance [48].

False-negative results may occur in the presence of extremely high levels of TG due to hook effect [48]. Specifically, two-site non-competitive IMAs are subject to the hook effect, as a result of a massive excess of analyte (antigen) that depletes the binding capacity of the capture antibody, leading to inappropriately normal or low values of analyte [45]. In the case of TG, a falsely low serum value may have important clinical consequences. Currently, commercially available TG assays are very resistant to this type of analytical interference, but it can still occasionally occur in patients with high-load metastatic disease (i.e., serum TG up to 1000 μg/L). Dilution of serum may be used to detect hook effects in suspicious cases [49]. In general, the concentration at which the hook effect can be excluded should be determined by the manufacturers and verified locally by each laboratory [48]. In addition, IMAs may be affected by paraproteins, heterophilic antibodies and high biotin serum concentration especially in streptavidin-biotin coupling assays [47]. The existence of antithyroglobulin antibodies (ATG) interferes with TG measurement, altering antigen-antibody complex formation resulting in a false-negative test in current IMAs [47]. The monitoring of both TG and ATG is fundamental during the follow-up of thyroid cancer patients [47] since 25% of DTC patients were positive for ATG [50]. Variations in serum ATG concentrations, determined longitudinally employing the same IMA, may be used as a surrogate tumor marker for residual or progressive thyroid cancer [48]. The trend of ATG levels is more important than the absolute level; indeed, a reduction in serum ATG concentrations is an indication of a disease-free condition. Conversely, the persistence or increase of ATG concentrations should suggest suspicion of persistent disease or recurrence [48]. Interestingly, liquid chromatography coupled to tandem mass spectrometry (LC-MS/MS) methods are less affected by the presence of autoantibodies. Thus, some authors suggest the use of LC-MS/MS for measuring TG serum levels in ATG-positive patients [51]. The use of trypsin digestion before the measurement results in the cleavage of all proteins, thus eliminating autoantibodies [52]. However, there is still much to be done towards the harmonization of LC-MS/MS [53]. In particular, LC-MS/MS still shows variances in the choice of the calibrator, although the latest methods show good agreement of results. For this reason, nowadays it is not the first-line test but is used only in selected cases [48].

Since the release of TG from both non-malignant thyrocytes and DTC cells is generally dependent on TSH, serum TG determination under TSH stimulation has long been considered the gold standard for ensuring remission 6–18 months after ablation of the malignant cells in addition to being recommended in many guidelines [22,28]. TSH stimulation can be achieved by T4 withdrawal (endogenous stimulation) or by recombinant human TSH (rhTSH) administration (exogenous stimulation). Both approaches provide high serum TSH concentration (>30 mIU/L) [54]. At such TSH levels, an unmeasurable TG suggests a very low risk of recurrence. Conversely, TG concentrations higher than 1–2 μg/L have to be considered suggestive of disease persistence or recurrence [54]. In recent years, the introduction of hsTG IMAs has reduced the need for stimulation of TSH to measure TG concentrations during initial and long-term follow-ups of patients with DTC [48,55,56]. In this regard, several studies were published on the role of basal hsTG versus rhTSH-stimulated hsTG showing that unstimulated serum TG concentrations lower than 0.2 μg/L ruled out additional stimulation tests in most cases [55,57,58,59,60,61]. Conversely, little is known about the role of basal hsTG versus endogenous stimulated hsTG [62,63]. In this respect, Trimboli et al. demonstrated that TG levels lower than 0.23 μg/L after T4 withdrawal, in low- and high-risk DTC patients, is an accurate marker of disease freedom [54]. In addition to its prognostic and predictive role of disease recurrence after total ablation, there has been an increased focus on the role of TG also in the time interval between surgery and RAI [64,65]. The value of TG as a predictive factor before treatment with ^131^I is controversial due to several factors including the existence of residual thyroid tissue after surgery [66,67], TSH concentration, and individual risk of loco-regional or remote metastases [68,69]. In addition, the correct timing for post-operative measurement of TG and its stimulated or suppressed cut-off postoperatively, which can confirm or exclude the presence of lesions, has not yet been identified [70]. In this context, in a recent study by Signori et al., hsTG was measured at three different time points in a selected population of patients operated for PTC [71], specifically after thyroidectomy but before RAI ablation (in euthyroidism, 40 days after surgery), at the time of RAI ablation (in hypothyroidism) and after RAI ablation (in euthyroidism). After a three-year follow-up, the results showed that the determination of hsTG before RAI therapy (first-time point) was a reliable prognostic indicator to predict future nodal or distant disease recurrence, useful to guide patient management.

### 3.2. Calcitonin

Human calcitonin (CT) is a 32-amino acid polypeptide hormone secreted mainly by the parafollicular cells of the thyroid gland and it is involved in calcium–phosphorous metabolism. Other tissues can produce CT including lungs, parathyroid glands, bladder, small intestine, liver and thymus [72].

CT was described for the first time in 1962 by Copp and Cheney as a modulator of calcium tone [73]. CT is synthesized as part of a larger prohormone, called procalcitonin (ProCT), a precursor peptide derived from pre-procalcitonin and, its secretion is primarily regulated by calcium and gastrin levels in serum [72].

In healthy subjects, CT levels are influenced by several factors including gender and age (slightly higher in men than in women and in the pediatric population than in adults), body mass index (BMI) and smoking [74]. Based on the method employed, at least 90% of healthy adults show serum CT concentrations below 10 ng/L and, among them, more than 50% below the limit of detection (LOD) of current IMAs [74,75].

Elevated serum CT levels are highly sensitive for the diagnosis of MTC in patients with nodular/multinodular goiter, but lack strong specificity [76]. In fact, several drugs can stimulate CT secretion, i.e., proton pump inhibitors (PPIs) and beta-blockers and, as with most tumor biomarkers, the blood concentration of CT may also increase in other pathological conditions such as chronic renal failure, autoimmune thyroiditis, hypergastrinemia, sepsis, type 1A pseudohypoparathyroidism and mastocytosis [76]. In addition, several types of neoplasms, including breast cancer and neuroendocrine neoplasms (NENs), may present with an ectopic CT secretion [77]. Therefore, the identification of a reliable cut-off for basal CT in order to support a more accurate initial diagnostic evaluation of thyroid nodules is still missing [78]. Giannetta et al., in an extensive review of the literature, suggested 100 ng/L as cut-off to strongly suspect thyroid malignancy, with a positive predictive value (PPV) for MTC reaching 100%, whereas in subjects showing moderately high values (10–100 ng/L) diagnostic accuracy could be enhanced by performing a stimulation test (e.g., Calcium test) [79]. Over the years, several authors have proposed various cut-offs for basal CT, generally below 100 ng/L with differences between females and males [78,80,81]. The discrepancies between the cut-offs in different studies can, at least in part, be attributed to the different population inclusion criteria and the different methods used for CT measurement [78]. Similarly, cut-offs for CT after stimulation vary in the different studies published with a distinction between females and males [78]. A slight CT increase is observed in C-cell hyperplasia (CCH), with concentrations setting between 10 and 20 ng/L. However, it is reported in a Cochrane systematic review that only 0.32% of patients with thyroid nodules were diagnosed with MTC, showing a low prevalence of the disease and opening a debate for routine CT measurement in these patients [82]. In the preoperative setting, basal CT levels are indicative of tumor burden, metastatic potential and lymph node invasion and extent [83]. Postoperatively, CT concentrations are of great prognostic value; basal CT should be measured 3 months after surgery and monitored every 6–12 months as suggested in the ATA guidelines [35].

Several analytical methods have been used to measure serum CT levels over the decades. They were first revealed by RIA, whose inaccuracy was attributable to the use of polyclonal antibodies that detected both mature and immature monomers of CT, as well as other circulating forms (precursors and degradation products) [84]. RIA was replaced by two-sided IRMA, whose improved specificity was derived from the implementation of two monoclonal antibodies, capable of binding to two different specific epitopes within the CT molecule [85]. The LOD reached 1 ng/L following the introduction of IMAs based on fluorescence and chemiluminescence, which were more sensitive and specific for CT in their architecture. Currently, the employment of an ECLIA based on streptavidin–biotin technology has shortened testing time and lowered the LOD to less than 1 ng/L [86].

The introduction of more accurate cut-offs would be helpful in the differential diagnosis between CCH and micro-MTC and the exclusion of ectopic CT production by various NENs [83]. CT levels measured with different commercial assays may vary widely, and it is of primary importance that each laboratory establishes and maintains its reference intervals and cut-offs; patients’ follow-up should be performed using the same method, and re-baseline is required in case of method changeover [87].

### 3.3. Carcinoembryonic Antigen

Carcinoembryonic antigen (CEA) is an intercellular adhesion glycoprotein, with a molecular weight of 200 kDa, initially detected in human tissue of colorectal cancer in 1965 by Gold and Freedman [88]. CEA is expressed in normal tissue and a broad range of epithelial neoplasms (e.g., colorectal cancer, lung cancer, pancreatic cancer, etc.) [89]. Since CEA is released into the bloodstream, the measurement of circulating CEA is used as a tool for early diagnosis, monitoring of cancer recurrence and treatment efficacy [90]. In healthy subjects, the blood concentration of CEA generally ranges from 2.5 to 5 ng/mL, with higher values in men than in women. CEA values greater than 5 ng/mL may indicate the presence of malignant tumors. However, as in the majority of tumor biomarkers, blood levels of CEA may also increase in non-neoplastic diseases such as ulcerative colitis, pancreatitis, liver cirrhosis, hepatitis, renal insufficiency and in heavy smokers [91]. In addition to cancer diagnosis and monitoring, there are currently several therapeutic approaches targeting this biomarker, such as in metastatic colorectal and lung carcinomas that are CEA-positive [92].

Literature reports that 60–70% of MTC patients have elevated serum CEA levels [93]. Although not specific, CEA can have a role as a marker of tumor progression and invasion, especially in MTC with low or no production of CT, as its blood levels are associated with tumor size, lymph node involvement and also distant metastases [93,94]. CEA is also useful for the evaluation of response to initial therapy in MTC patients [93]. Some studies have considered CEA as a marker of dedifferentiation in MTC follow-up. In detail, in cases of decreased levels of postoperative CT, increased levels of CEA may be an indication of dedifferentiation [95]. A fraction of MTC shows no immunohistochemical expression of CT and low or negative serum levels of both CT and CEA. This subset of MTC enters into differential diagnosis of thyroid high-grade NENs, which are also negative for CT and CEA both at tissue and blood levels [96].

Currently, the detection of CEA is performed by automated CLIA which has almost completely displaced RIA and enzyme-linked immunosorbent assay (ELISA) [97,98]. Although the International Reference Preparation (IRP) has been in use for several decades, numerous studies have reported the persistence of method- and/or manufacturer-dependent differences for CEA [99,100,101]. Over the years, there has been a clear improvement in the maximum bias among manufacturers, from 85% in 2005 [102] to less than 50% in 2023 [103]. There was also an improvement within the same method with intra-assay coefficients of variation (CV) less than 10% [103]. However, despite technological improvements over the last two decades, a very recent study comparing six immunoassays has shown that there is still no full harmonization for CEA determination [98]. The lack of inter-method comparability may be due to several factors [103]. Firstly, structural aspects (i.e., high molecular weight, significant carbohydrate content, several isoforms and numerous possible epitopes) may influence the definition of specific peptide epitopes suitable for antibody binding in IMAs [103]. Secondly, antigen–antibody binding affinity may vary depending on the antibodies used in the assay as well as the conformation and glycosylation of their epitopes [104,105]. Finally, the characteristics of antibodies may affect the specificity of the binding, which is probably lower for polyclonal than monoclonal antibodies [106]. Therefore, in the absence of a reference method and complete harmonization between IMAs, each laboratory must establish its reference intervals/cut-offs to reflect the situation of its specific population. Moreover, the results of the CEA have to be always evaluated together with the patient’s medical history, clinical examination and imaging information [98].

### 3.4. Carbohydrate Antigen 19-9

Recently, carbohydrate antigen 19-9 (Ca 19-9) has emerged as a potentially useful prognostic predictor in both MTC and advanced DTC [107]. Ca 19-9 is a 36 kDa glycolipid belonging to the mucin family. Mucins are highly glycosylated proteins, abundantly distributed on the surface of epithelial cells that present alterations in their expression and structure in numerous pre-neoplastic and neoplastic lesions [108]. Serum levels of Ca 19-9 are high in carcinomas of the digestive tract, especially pancreatic, but also in lung, ovarian and uterine tumors [109,110]. Unfortunately, the lack of satisfactory sensitivity and especially specificity does not allow its measurement for early diagnosis of cancer [108,111]. Currently, it is used in the follow-up phase, particularly in the monitoring of pancreatic cancer [109].

Several studies, published in the last 10–15 years, have identified Ca 19-9 as a marker of MTC, expressed both at tissue and blood levels [112,113]. Overall, these results showed that increased serum Ca 19-9 levels are an adverse prognostic factor in patients with advanced MTC, especially in cases with a higher risk of short-term mortality [114,115]. Similarly, Alencar et al. reported that serum Ca 19-9 may have a role as a prognostic factor in patients with MTC [116]. On the other hand, the precise relationship between PTC and Ca 19-9 in tissues and serum has not been well established. Some studies have shown positivity for Ca 19-9 immunohistochemical staining in tumor tissue [117,118,119]. Little detailed information is available on serum Ca 19-9 levels in patients with PTC. Kihara et al. reported a case of hepatic metastasis in PTC patients after several years from thyroidectomy accompanied by elevated serological levels of Ca 19-9. After partial liver resection, a significant decrease in serum concentrations of Ca 19-9 was observed [120]. In addition, Yamaguchi et al. described a case of elevated serum levels of Ca 19-9 in a patient with PTC-related lung metastasis diagnosed 15 years after thyroidectomy [118]. Finally, very recently, Kihara et al. have retrospectively analyzed 196 patients with PTC (maximum diameter 2 cm). For each patient, serum Ca 19-9 values were determined before and after the surgery. Elevated serum levels of Ca 19-9 before thyroidectomy were observed in 6.1% of patients. After the surgical procedure, serum levels of Ca 19-9 in all patients decreased back to the normal range. Although further studies with longer follow-up are needed, the authors suggested serum Ca 19-9 levels as a tumor marker for PTC [121].

To date, most IMAs for quantitative detection of Ca 19-9 use a sandwich format and depend on the use of the monoclonal antibody 1116-NS-19-9, called Centocor, which recognizes the sialyl Lewis A glycan motif, a member of the Lewis family of blood group antigens involved in the binding of glycans, lipids and proteins [122,123]. Although extensive research has been conducted on the determination of Ca 19-9, challenges remain in achieving standardization and harmonization between methods. Recent automation of IMAs has certainly improved accuracy, but it has not yet succeeded in reducing the discrepancy between results obtained from the same samples using different methods [124,125,126,127]. In this respect, the Society for Promoting Quality Assurance in Medical Laboratories (INSTAND, Germany) observed a manufacturer-dependent bias of up to 194% for Ca 19-9 as part of the results of the external quality assessment (EQA) in 2005 [102]. Significant variations have also been reported in clinical trials comparing different manufacturers [128]. Recently, Kremser et al. conducted a longitudinal re-evaluation of EQA data for some cancer markers including Ca 19-9 [129]. The authors compared intra- and inter-method variations between participants using the most common analytical platforms and tested their adherence to EQA limits [129]. They concluded that the intra-method precision of most analytical platforms has become accepted for Ca 19-9 (CV less than 16% for each individual collective) [129]. Conversely, the variability between different methods remains significant [129]. Potential causes of these differences may include the use of monoclonal antibodies with different antigen-binding sites, antigen modifications and different assay architectures/formulations [129].

Finally, tissue expression dependence and circulating levels of sLeA in Lewis blood group also influence the sensitivity of Ca 19-9 assay. False negative results were found in subjects with a negative Lewis genotype, which represents 5–10% of the Caucasian population, while no data are available on other ethnicities [110]. Interestingly, low or medium levels of Ca 19-9 (approximately 100 kU/l) have been reported in some patients with negative Lewis genotype and advanced pancreatic cancer [122,130,131].

The above-cited biomarkers are summarized in Table 1.

## 4. Emerging Blood Biomarkers in Thyroid Cancer

**Figure 1:**
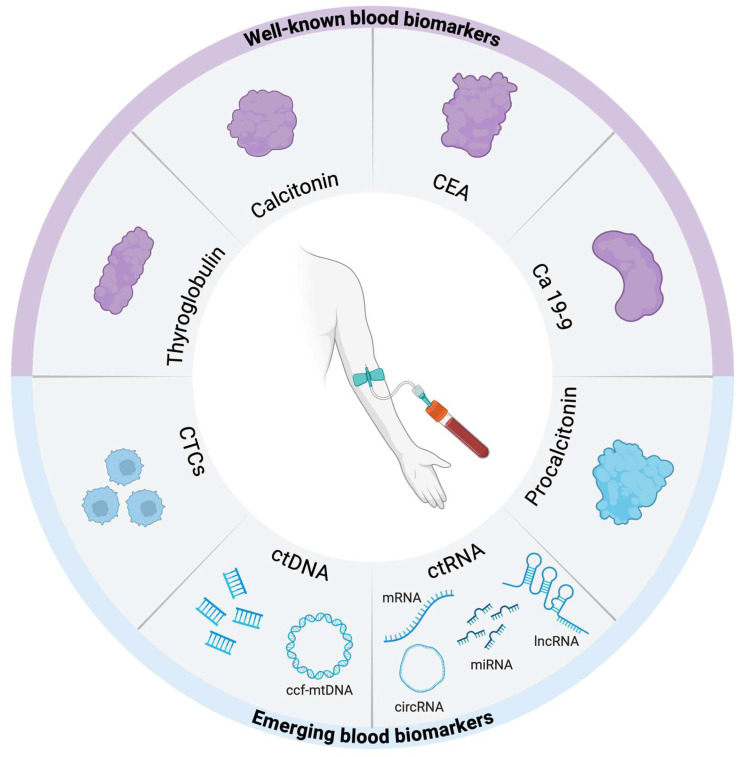
Overview of blood biomarkers in thyroid cancer. Schematic representation of well-known and emerging biomarkers for thyroid tumors. CEA, carcinoembryonic antigen; Ca 19-9, cancer antigen 19-9; CTCs, circulating tumor cells; ctDNA, circulating tumor DNA; ccf-mtDNA, circulating cell-free mitochondrial DNA; ctRNA, circulating tumor RNA; mRNA, messenger RNA; miRNA, microRNA, lncRNA, long non-coding RNA; circRNA, circular RNA. Created with BioRender.com accessed on 16 February 2025.

Besides the well-known thyroid tumor biomarkers, new circulating biomarkers are now emerging. Advances in genomic, transcriptomic and proteomic technologies have allowed the development of novel tumor biomarkers. Liquid biopsy is a minimally invasive laboratory test that permits the detection and analysis of circulating tumor cells (CTCs) and circulating tumor nucleic acids (ctNAs) in the peripheral blood and other body fluids of patients with cancer, offering real-time information on tumor diagnosis, progression and therapeutic response. In this section, we have reviewed the recent discoveries on thyroid biomarkers presenting promising clinical uses for diagnosing and following up on thyroid disease (Figure 1).

### 4.1. Circulating Tumor Cells

CTCs are tumor cells derived from the primary solid tumor that extravasate into and circulate mainly in the bloodstream [132]. CTCs possess significant metastatic potential thanks to their capability to reach other sites easily since their presence in the blood [133]. However, only a small percentage of CTCs can metastasize, suggesting that escaping the immune system and interacting with the specific microenvironment in the secondary loci are required [134,135].

With the development of molecular technologies, CTCs isolation and enrichment have allowed a detailed investigation into CTCs biology, thus providing greater details about tumor gene mutations and heterogeneity. Nowadays, CTCs isolation and identification are based on antigen-dependent (such as epithelial marker, e.g., EpCAM) [136] or antigen-independent approaches combined with molecular techniques [137]. Indeed, thanks to single-cell sequencing technology, CTCs genome and transcriptome have been extensively investigated [138,139]. Importantly, CTCs may facilitate clinical practice. Indeed, many clinical trials have been carried out focusing mainly on the usage of CTCs in breast and prostate cancer considering clinical prognosis and therapy response [140]. Recent data have highlighted their potential not only in early cancer detection [141,142], but also in the minimal residual disease and the relapse of the tumor [143].

Few studies have been conducted to point out new potential alternative biomarkers for clinical application in thyroid cancer patients considering the diagnostic value of CTCs. Li et al. demonstrated a higher detection of CTCs in patients affected by PTC and by FTC associated with a shorter overall survival. Moreover, the authors showed that increased CD133 levels were associated with the differentiation grades of thyroid cancers [144]. A clear correlation was found between the number of CTCs and the tumor stage in PTC, FTC, and MTC patients. Moreover, compared to the control subjects, CTCs were detectable in patients with DTC after complete thyroidectomy in the absence of serum TG and no evidence of tumor recurrence. Furthermore, the number of CTCs correlated to the radioiodine therapy in PTC patients. The authors suggest that PTC patients need a restrictive follow-up due to the increased numbers of CTCs even in a remission condition [133]. A similar result has been obtained by Sriramareddy et al., who demonstrated the presence of CT-positive CTCs in the bloodstream of MTC patients following complete thyroidectomy although serum CT was not detectable. The authors encourage a strict follow-up for these patients since the number of CTCs was correlated with the probability of tumor relapse [145]. Indeed, Weng et al. demonstrated a positive correlation between the detection of CTCs and the metastasis rates in patients with PTC and with FTC correlated with poor progress-free survival [146]. Furthermore, Xu et al. proved a negative association between the overall survival and the number of CTCs in patients with metastatic MTC [147]. Additionally, a prospective study demonstrated the increased detection of CTCs mainly in patients with DTC, especially in those with metastases compared to healthy subjects [148]. However, a retrospective study concluded that the efficacy of CTCs in diagnosing thyroid cancer is still limited considering the number of CTCs and the antibodies against thyroid peroxidase (ATPO) values in a cohort of thyroid tumor patients divided into malignant and benign groups [149]. Nevertheless, a recent meta-analysis showed that CTCs expressing thyroid-stimulating hormone receptor (TSHR), rather than EpCAM, are a reliable marker for the diagnosis of patients with thyroid cancer recurrence or metastasis [150].

Interestingly, a recent study considered Survivin gene expression among different markers of malignancy. An increased Survivin mRNA isolated from mononuclear cells is indicative of low differentiation grades of thyroid cancers [146]. Similar results were reported by Li et al. In detail, CTCs were detected and CK19, Survivin and TG mRNAs were analyzed. The authors demonstrated that CTCs were increased in PTC patients with distant metastasis and highlighted the role of CTCs signature Survivin as a potential marker of PTC diagnosis [151].

### 4.2. ctNAs

On the other hand, ctNAs are cell-free nucleic acids, comprising circulating tumor DNA (ctDNA) and RNA (ctRNA), that originate from tumor cells and are shed into the blood circulation [152]. ctNAs can be released directly from the primary tumor, CTCs or tumor extracellular vesicles, thus harboring the mutational status of the original tumor [153,154,155]. ctDNAs are fragmented DNA [156], while ctRNAs include messenger RNA (mRNA) and non-coding RNAs (ncRNAs), such as microRNA (miRNAs), circular RNA (circRNA), and long non-coding RNA (lncRNA) [157]. The ncRNAs are functional RNA molecules that are not translated into a protein and play a key regulatory role in multiple biological functions. Indeed, their dysregulations have been implicated in multiple diseases including tumorigenesis acting as oncogenic molecules or tumor suppressors [158,159]. CtNAs analysis consists of the extraction of cell-free nucleic acids mainly from a blood sample, avoiding invasive procedures. Thus, the evaluation of ctNAs provides important information about the mutational spectrum of the tumor. As with CTCs, the development of NGS techniques and molecular technologies for the isolation of ctNAs have allowed us to deepen our knowledge of the characteristics of blood ctNA markers for the diagnosis, monitoring and prognosis of cancer [160]. However, there are some limitations, such as the low signal-to-noise ratio and the short bioavailability [152]. Therefore, the current challenge aims to improve the current expertise in using ctNAs as blood clinical markers as a tumor screening tool and for the detection of minimal residual disease.

#### 4.2.1. ctDNAs

The analysis of ctDNAs can detect the mutations and epigenetic changes useful for diagnosis and treatment strategy choice. In the thyroid cancer field, recent evidence observed the presence of ctDNA containing BRAFV600E mutation, the most frequent genetic event in DTC, in patients affected by PTC compared to those with benign nodules [161]. Moreover, a hypermethylation state of SLC5A8 and SLC26A4, associated with BRAFV600E mutation, was found in PTC patients [162]. Interestingly, the level of circulating BRAFV600E has been observed to dramatically decrease after surgery in PTC patients [161]. In addition, the detection of residual circulating BRAFV600E post-surgery is suggestive of recurrence in patients with PTC [163]. Circulating RETM918T mutation was detectable in the plasma of patients affected by MTC, suggesting a worse outcome [164]. However, it has been shown that there is a very low concordance in BRAF, KRAS, NRAS and TERT promoter mutations between primary or metastatic thyroid tissues and plasma ctDNAs in early stage thyroid cancer patients [165]. Nevertheless, through NGS analysis on ctDNAs extracted from blood samples of patients affected by different thyroid tumor types, it has been observed that the large majority of patients presented one or more genomic alterations and TP53 mutation was the most frequent in all thyroid tumor types, followed by BRAFV600E, RAS, RET, ALK and NTRK with variable frequency according to the type of tumors taken into account [166].

A mention of mitochondrial DNA (mtDNA) deserves to be discussed. The mtDNA is a double-stranded circular chromosome located in the mitochondria organelles in a tissue-specific number of copies [167]. The mitochondrial genome consists of 16 kb and contains genes that encode for its tRNAs and rRNAs and proteins involved in oxidative phosphorylation [168]. The involvement of mtDNA in the pathogenesis of several diseases including cancer has been demonstrated [169]. Indeed, tumors exhibit an altered bioenergetic process due to gene copy numbers or gene expression modifications [169]. For instance, an aberrant accumulation of mitochondria due to mtDNA mutations is characteristic of the oncocytic phenotype in thyroid gland tumors [170]. Moreover, an increased tissue mtDNA copy number is associated with carcinogenesis in PTC [171]. The altered presence of circulating cell-free mtDNA (ccf-mtDNA) is indicative of mitochondrial dysfunction and thus of a pathological condition [172]. A lower content in plasma ccf-mtDNA has been shown in patients with PTC compared with healthy subjects [173]. Thus, the ccf-mtDNA may be a valid alternative to ctDNA as it is easily detectable due to its abundance compared to ctDNA in the bloodstream and especially due to its strict correlation with tumor progression.

#### 4.2.2. ctRNAs

In the last decade, ncRNAs have yielded great interest as potential circulating biomarkers, particularly in cancer research. In contrast to ctDNAs, circulating ncRNAs can be easily quantified in the blood, especially miRNAs. Indeed, miRNAs reflect the clinical features of the tumor and their blood levels can be associated with treatment response and patient outcome [174]. To date, certain studies have been conducted on the expression profile and clinical significance of circulating miRNAs in thyroid cancer.

Using a microarray approach, Romeo et al. identified miR-375 as the most upregulated miRNA in C cells and plasma of MTC patients compared to healthy controls and subjects in remission, associating miR-375 with reduced overall survival and poor prognosis [175]. The same results were obtained by Censi et al., who showed serum miR-375 overexpression in pre-surgical MTC patients compared to controls and patients affected by different diseases. Unfortunately, no correlation was identified between serum and tissue. However, a concordance was observed between serum miR-375 and CT [176]. This data was also confirmed by Melone et al., who identified a serum molecular signature in MTC patients applying miRNome profiling. The authors reported an upregulation of miR-375, miR-144-3p, miR-7-5p and miR-335-5p [177]. MiR-144 was also detected by Shabani et al. Indeed, increased plasma levels of miR-144 and miR-34a were observed in MTC patients compared to controls. Interestingly, MTC patients carrying RET mutation presented a much higher expression of miR-144 and miR-34a than wild-type RET MTC patients [178]. Among miRNAs identified using miRNA arrays, plasma miR-26b-5p and miR-451a were observed to be highly expressed in a cohort of MTC patients. Interestingly, their expression decreased after surgery [179]. Furthermore, serum levels of miR-222-3p and miR-17-5p were found significantly increased in MTC patients compared to benign nodule and control groups. In the same study, the authors observed also a trend for patients affected by PTC. In detail, miR-222-3p, miR-17-5p, and miR-451a were shown to increase, whereas miR-146a-5p, miR-132-3p, and miR-183-3p were decreased in the serum of PTC patients and those with benign nodules compared to the control group [180]. Similar findings have resulted from using miRNA profiling that identified serum miR-221-3p, miR-222-3p, miR-146a-5p, miR-146b-5p, miR-24-3p, miR-191-5p, miR-103-3p and miR-28-3p as upregulated in PTC patients compared to healthy subjects. Among these miRNAs, miR-146a-5p, miR-221-3p and miR-222-3p markedly decreased after tumor excision in PTC patients. Moreover, levels of miR-146a-5p and miR-221-3p correlated with serum TG levels [181]. Yu et al. also demonstrated that the expression of serum miR-222, miR-151-5p and let-7e was higher in PTC patients compared to benign cases and healthy controls. In addition, a lower expression of miR-151-5p and miR-222 was shown in a subset of PTC patients after thyroidectomy [182]. Equally, plasma miR-222 and miR-146b were increased in pre-surgery PTC patients in comparison with healthy volunteers and after thyroidectomy. Importantly, Lee et al. demonstrated that miR-222 and miR-146b are associated with PTC recurrence [183]. Razei et al. evaluated miRNAs pre- and post-surgery and showed a decreased expression of plasma miR-222 and miR-181a in PTC patients after thyroidectomy. In addition, miR-181a and miR-146a distinguished between cancerous and benign cases. Moreover, the levels of miR-181a were associated with increasing tumor size in PTC cases. Interestingly, there was a correlation between miR-222 and BRAFV600E mutation in PTC patients [184]. Likewise, serum miR-221, miR-222, miR-31, and miR-151-5p were observed to decrease in PTC after surgery compared to PTC pre-surgery patients. Moreover, the serum amount of miR-222, miR-31, miR-151-5p and let-7 was revealed to increase, whereas miR-21 was decreased in PTC patients relative to controls and patients affected by benign nodules [185]. Graham et al. showed an mRNA profiling to distinguish PTC from benign nodules. Particularly, serum miR-146a-5p and miR-199b-3p were downregulated, whereas let7b-5p and miR-10a-5p were upregulated in PTC serum samples than benign tumor [186]. The level of serum miR-579, miR-95, miR-29b, and miR-190 were lower in PTC patients respect to controls and patients with nodular goiters, among which miR-95 and miR-190 resulted the most promising [187]. An increased expression of plasma miR-25-3p, miR-451a, miR-140-3p and let-7i was observed in PTC cases compared to benign nodules or healthy controls. Importantly, miR-25-3p and miR-451a decreased after tumor excision. Moreover, the plasma levels of miR-25-3p and miR-451a were correlated with those expressed in thyroid tissues from PTC patients [188].

In summary, miR-375 and miR-144 turned out to be the most interesting miRNAs dysregulated in plasma and serum of MTC patients, whereas miR-222, miR-221, miR-146a, miR-151, miR-31, and miR-21 in PTC cases. Thus, these miRNAs are promising circulating biomarkers for the management of thyroid tumor disease (Table 2).

In the literature, there is very little evidence of the presence of lncRNAs in the blood of thyroid cancer patients. For instance, Jiang et al. identified 6 lncRNAs (CCAT1, SYNPR, SFTA1P, HOTAIR, HCG22, and CLDN10) in the plasma of PTC patients. Furthermore, all these lncRNAs correlate with overall survival affecting progression and invasion in thyroid tumors. Moreover, CCAT1, SYNPR, SFTA1P, HOTAIR, and HCG22 were found to be upregulated in PTC tumor tissue except for CLDN10, which was downregulated (Table 2) [189]. Other authors demonstrated the involvement of lncRNAs in thyroid cancer tissue, but not their presence in the bloodstream. Nevertheless, the lncRNAs MALAT1 [190], HOTAIR [191], and BANCR [192] are encouraging candidates for circulating thyroid cancer blood markers.

Nothing is known about circulating circRNAs’ implication in thyroid tumors. However, circRNAs are emerging as promising biomarkers for thyroid cancer. Indeed, there is evidence of the involvement of circRNAs in the onco-pathogenesis of the thyroid. For instance, it has been demonstrated that the impairment of circ-ITCH/miR--22-3p/CBL/β-catenin axis in PTC development and progression. In detail, circ-ITCH competes with miR--22-3p to upregulate the expression of CBL, thus inactivating the Wnt/β-catenin pathway and consequently attenuating PTC progression [193]. Yao et al. identified circ0058124 as a novel driver for PTC tumorigenesis by regulating NUMB through binding to miR-218-5p, thus repressing NOTCH3/GATAD2A signaling [194]. Likewise, circZFR plays a role in PTC cell proliferation, migration and invasion by miR-1261/C8orf4 axis [195] and circNUP214 acts as an oncogene and sponges miR-145 and its target ZEB2 in PTC cells [196]. Finally, circ-0004458 was found to be overexpressed in PTC tissues and cells. Circ-0004458 silencing induced cell cycle arrest and apoptosis by miR-885-5p/RAC1 pathway [197].

Regarding coding RNAs, Yang et al. have developed a multiplex approach for the quantification of circulating transcripts in thyroid tumor patients, identifying 4 circulating RNAs (thyroid peroxidase TPO, TG, glial cell line-derived neurotrophic factor family receptor alpha-2 GFRA2, and iodotyrosine deiodinase IYD) in the plasma of thyroid cancer patients, among which TPO transcript resulted in the most promising marker to estimate residual disease [198]. Similar results were obtained in two different studies whereby the levels of TG mRNA were estimated in the peripheral blood of thyroid tumor patients [199,200]. In detail, it has been demonstrated that the presence of TG transcript in blood samples of subjects affected by PTC [200] and in all metastatic DTC cases [199]. Nevertheless, TG mRNA was detectable in patients with benign thyroid nodules as well as in healthy subjects [199], potentially derived from lymphocytes and renal cells besides circulating thyrocytes [201,202]. Among well-known thyroid-associated markers, it has been suggested that the detection of TSHR mRNA in patients with thyroid lesions be used not only as a marker of recurrence but also as a marker of diagnosis and aggressiveness [203,204,205,206,207]. Likewise, it has been demonstrated that the levels of blood CT-related polypeptide alpha transcript (CT-CALCA) correlated with serum CT in MTC patients, including RET mutation carriers [208]. Finally, Lubitz et al. demonstrated the detection of BRAFV600E mutation in reverse-transcribed RNA isolated from peripheral blood lymphocytes of PTC patients [209].

Extracellular vesicles (EVs) deserve specific attention, in particular the well-characterized exosomes. Over the last years, many authors have reported the involvement of exosomes in tumor progression and metastasis processes. In detail, exosomes are extracellular vesicles secreted from cells carrying nucleic acids (such as miRNAs, circRNAs and lncRNAs), proteins, lipids, and metabolites [210]. Recently, evidence highlighted the dysregulation of exosome content in the pathogenesis of thyroid cancer, revealing their potential as biomarkers in tumor diagnosis and clinical prognosis. Among the different exosomal miRNAs, miR24-3p, miR146a-5p, miR181a-5p and miR382-5p were found to be downregulated, whereas miR127-3p and miR376a-3p were upregulated in the serum of PTC patients compared to healthy subjects. Interestingly, exosomal miR24-3p was positively correlated with the same circulating miRNA free of any encapsulation [211]. Similarly, Liang et al. observed a lower expression of plasma exosomal miR-16-2-3p, miR-34c-5p, miR-182-5p, miR-146b-5p, miR-223-3p and miR-223-5p in nodular goitres and PTC patients compared to controls. Moreover, the authors proposed miR-16-2-3p and miR-223-5p as biomarkers to be utilized to distinguish between benign and malignant nodules [212]. A decreased serum exosomal miR-29a was also identified in PTC subjects relative to controls [213]. Conversely, increased expression of plasma or serum EVs-derived miR-1-3p, miR-206, miR-221-3p [214], miR-10a-5p, miR-34a-5p, miR-346 [215], miR-145 [216], miR-376a-3p, miR-485-3p, miR4306 and miR-4433a-5p [217] well-differentiated PTC patients from controls [214,215,216,217], benign thyroid nodules from malignant ones [217] and after surgery [214]. Besides PTC cases, Samsonov et al. observed overexpression of miR-21, miR-31, miR146a, miR-181a, and miR-221 in plasma exosomes of different thyroid tumor cases compared to normal subjects. Interestingly, miR-21, miR126, and miR145 distinguish between PTC and benign tumor patients, whereas miR-31 between FTC and adenomas. In the same study, miR-21 and miR-181a were able to differentiate between FTC and PTC patients [218]. Regarding the possibility of improving prognosis, plasma exosomal miR146b-5p and miR222-3p were identified as upregulated in PTC patients with lymph node metastasis (LNM) compared to those without invasion [219]. Similar results were obtained by Chen et al., who identified plasma exosomal miR-6774-3p and miR-6879-5p as discriminants among PTC patients with and without LNM [220]. In addition to miRNAs, other ncRNAs are found to be dysregulated in thyroid patients’ exosomes. Within circRNAs, circ-007293 [221,222], circ-031752 and circ-020135 [222] were upregulated in PTC patients’ serum, whereas among lncRNAs, DOCK9-AS2 was enriched in exosomes derived from PTC patients’ plasma [223]. Interestingly, some authors provided evidence that exosome-derived proteins are dysregulated in thyroid cancer as well as ncRNAs. Indeed, through mass spectrometry, a different pattern of ITGB2, TLN1, CAPNS1 and SRC in exosome-derived serum from PTC patients has been identified, correlating their increased expression with LNM [224]. Furthermore, using the same approach, bone marrow stromal cell antigen 2 (BST2) was found to be well associated with PTMC progression [225]. An enhanced expression was also shown for chaperone proteins Hsp27, Hsp60, and Hsp90 in thyroid tissue and plasma exosomes of PTC patients as compared with benign goitre and after thyroidectomy [226]. Finally, a recent multi-omics analysis pointed out a transcriptomic and proteomic signature for indeterminate thyroid nodules. In detail, the authors revealed an enhanced expression of EV-derived CXCR7, CD147, SDC4 and EpCAM in the plasma of patients with indeterminate thyroid nodules compared with healthy controls. Additionally, mir-195-3p was found to be upregulated and mir-3176, mir-205-5p, novel-hsa-mir-208-3p, mir-3529-3p and let-7i-3p downregulated [227]. Thus, exosomes are key players in thyroid cancer pathogenesis and therefore can be considered promising tumor biomarkers on a par with ctNAs (Table 2).

**Table 2:** List of promising extracellular vesicles-derived and free circulating ncRNAs for the management of thyroid tumor disease with potential diagnostic and prognostic significance. In the table, the expression level is indicated as follows: upregulated ↑ and downregulated ↓. PTC, papillary thyroid carcinoma; FTC, follicular thyroid carcinoma; MTC, medullary thyroid carcinoma.

Circulating ncRNA	Thyroid Tumor	Expression Level	Biological Matrix	References
miR-1	PTC	↑	Plasma-derived exosomes	[214]
miR-7	MTC	↑	Serum	[177]
miR-10	PTC	↑	Serum, Plasma-derived exosomes	[186,215]
miR-16	PTC	↓	Plasma-derived exosomes	[180]
miR-17	MTC, PTC	↑	Serum	[180]
miR-21	FTC, PTC	↑↓	Serum, Plasma-derived exosomes	[185,218]
miR-24	PTC	↑↓	Serum, Serum-derived exosomes	[181,211]
miR-25	PTC	↑	Plasma	[188]
miR-26	MTC	↑	Plasma	[179]
miR-28	PTC	↑	Serum	[181]
miR-29	PTC	↓	Serum, Serum-derived exosomes	[187,213]
miR-31	FTC, PTC	↑	Serum, Plasma-derived exosomes	[185,218]
miR-34	MTC, PTC	↑↓	Plasma, Plasma-derived exosomes	[178,212,215]
miR-95	PTC	↓	Serum	[187]
miR-103	PTC	↑	Serum	[181]
miR-126	PTC	↑	Plasma-derived exosomes	[218]
miR127	PTC	↑	Serum-derived exosomes	[211]
miR-132	PTC	↓	Serum	[180]
miR-140	PTC	↑	Plasma	[188]
miR-144	MTC	↑	Plasma, Serum	[177,178]
miR-145	PTC	↑	Plasma/Serum-derived exosomes	[216,218]
miR-146 family	PTC	↑↓	Plasma, Serum, Plasma/Serum-derived exosomes	[180,181,183,184,186,211,212,218,219]
miR-151	PTC	↑	Serum	[182,185]
miR-181	FTC, PTC	↑↓	Plasma, Plasma/Serum-derived exosomes	[184,211,218]
miR-182	PTC	↓	Plasma-derived exosomes	[212]
miR-183	PTC	↓	Serum	[180]
miR-190	PTC	↓	Serum	[187]
miR-191	PTC	↑	Serum	[181]
miR-199	PTC	↓	Serum	[186]
miR-206	PTC	↑	Plasma-derived exosomes	[214]
miR-221	PTC	↑	Serum, Plasma-derived exosomes/EVs	[181,185,214,218]
miR-222	MTC, PTC	↑	Plasma, Serum	[180,181,182,183,184,185,219]
miR-223	PTC	↓	Plasma-derived exosomes	[212]
miR-335	MTC	↑	Serum	[177]
miR-346	PTC	↑	Plasma-derived exosomes	[186,215]
miR-375	MTC	↑	Plasma, Serum	[175,176]
miR376	PTC	↑	Plasma/Serum-derived exosomes	[211,217]
miR382	PTC	↓	Serum-derived exosomes	[211]
miR-451	MTC, PTC	↑	Plasma, Serum	[179,180,188]
miR485	PTC	↑	Plasma-derived exosomes	[217]
miR-579	PTC	↓	Serum	[187]
miR4306	PTC	↑	Plasma-derived exosomes	[217]
miR4433	PTC	↑	Plasma-derived exosomes	[217]
miR6774	PTC	↑	Plasma-derived exosomes	[220]
miR6879	PTC	↑	Plasma-derived exosomes	[220]
let-7 family	PTC	↑	Plasma, Serum	[182,185,186,188]
circ-007293	PTC	↑	Serum-derived exosomes	[221,222]
circ-031752	PTC	↑	Serum-derived exosomes	[222]
circ-020135	PTC	↑	Serum-derived exosomes	[222]
CCAT1	PTC	↑	Plasma	[189]
SYNPR	PTC	↑	Plasma	[189]
SFTA1P	PTC	↑	Plasma	[189]
HOTAIR	PTC	↑	Plasma	[189]
HCG22	PTC	↑	Plasma	[189]
CLDN10	PTC	↓	Plasma	[189]
DOCK9-AS2	PTC	↓	Plasma-derived exosomes	[223]

### 4.3. Procalcitonin

Procalcitonin (ProCT) is the peptide precursor of CT, a hormone synthesized by the parafollicular C cells of the thyroid and involved in calcium homeostasis [228]. ProCT is also released by the neuroendocrine cells of the lung and intestine in response to inflammatory stimuli [228]. ProCT was first described as a marker of sepsis (differentiating bacterial from non-bacterial infection) and multiple organ failure as well as to manage antibiotic therapy [229]. Besides CT as a well-known marker for MTC, there has been a shift in focus on ProCT as an emerging blood biomarker [230,231]. Indeed, ProCT has a half-life of 24 h and is less influenced by circadian rhythm compared to CT [232,233,234]. Moreover, ProCT is more stable at room temperature than CT and presents a comparable measurement between different current commercial IMAs [93]. However, ProCT is influenced by trauma and the inflammatory state of the patient, thus limiting its use in clinical practice in the case of inflammatory conditions. For instance, similarities between ProCT and CT values have been observed reflecting the clinical states of patients with MTC using Roche ECLIA for CT measurement and Roche, PES, Abbott for ProCT [235]. Similar results were obtained in other studies [236,237,238,239], demonstrating that ProCT presents an equivalent or even superior alternative to CT for the follow-up of MTC patients. A recent meta-analysis showed how serum ProCT can be considered a highly accurate test for MTC management, in terms of diagnosis and disease monitoring, thanks to its higher sensitivity and specificity [240]. In particular, using a fully automated homogeneous TRACE immunometric fluorescent assay on the Kryptor^®^ system, a cut-off value of ProCT > 0.1 ng/mL is considered a good marker for the diagnosis of MTC since it resulted in a sensitivity of 100%, specificity of 99.9%, PPV of 77.8%, and NPV of 100% [241]. Furthermore, the same authors suggest measuring ProCT to detect false hypercalcitoninemia due to heterophilic antibody interference [242]. Moreover, a study conducted by Kratzsch et al. demonstrated the usage of ProCT as an alternative method to CT to overcome false hypercalcitonemic conditions, in this case associated with PPIs therapy, chronic kidney disease and Hashimoto’s thyroiditis. In this study, CT was tested using two fully automated assays (IMMULITE, Siemens Healthineers and Liaison, Diasorin) and one nonautomated assay (IRMA, Medipan), and ProCT using Brahms Kryptor (ThermoFisher Scientific) [243]. Furthermore, the possibility to measure both CT and ProCT markers on the same automated platform facilitates the interpretation of the laboratory results by matching reflex strategies.

## 5. Discussion

This review explores the current literature data to critically analyze the benefits and limitations of routinely measured circulating biomarkers for the diagnosis and/or monitoring of DTC and MTC. The review also sheds light on new circulating biomarkers, focusing on the challenges of their use in the clinical management of thyroid cancer.

Thyroid cancer is the most frequent endocrine neoplasm. Its incidence rate is rapidly increasing worldwide [7], representing a potential threat to public health [244]. In fact, although having a good prognosis in a large percentage of cases, thyroid cancer can evolve negatively with lymph node invasion and distant metastasis [245].

CT and TG were the first two biomarkers discovered almost 50 years ago [1,2,246]. TG is the best available tumor marker for DTC after (near-) total thyroidectomy and subsequent RAI ablation of the remaining thyroid tissue [31]. Its periodic measurement provides crucial information on the patient’s response to treatment and the patient’s status regarding a possible recurrence of the disease after successful treatment [31], in association with the use of proper imaging procedures [247]. However, the determination of TG, as described before, is affected by the presence of antibodies against TG that negatively interfere with the measurement. Not being affected by the presence of any interfering antibodies, LC-MS/MS has been suggested as a second-line test in some selected cases. In addition, very recently, the estimation of TG transcript levels in peripheral blood has also been proposed as an innovative approach for overcoming antibody-mediated interference [173,174]. However, the presence of transcript variants, the mRNA expression from other sources, and the setting up of the PCR (such as primer design and number of amplification cycles) need to be improved. All these aspects can lead to false-negative or false-positive screening results. On the other hand, CT is used as a screening test in multinodular goiter allowing earlier MTC diagnosis and thus earlier intervention and higher cure rates of the disease [35]. Despite these observations, international scientific societies do not recommend either for or against serum CT determination as a screening test [17,28] since concerns related to cost-effectiveness and the association of high CT serum levels with diseases other than MTC continue to be discussed [41]. For example, CCH is a common cause of marginally elevated CT preceding tumor development in familial MTC. However, increased CT is also found in PTC, autoimmune thyroiditis, chronic renal failure, and non-thyroid-related conditions such as smoking or the use of PPIs. Finally, CT may also be elevated in patients with NENs [41]. Moreover, CT-negative tumors with aggressive biological behavior have been described [36]. In these cases, other markers such as ProCT and CEA may be useful [35].

Currently, serum levels of TG, CT, ATG, CEA and Ca 19-9 are measured mainly by IMAs, which are based on antigen-antibody reactions. Although they display numerous advantages, IMAs are sensitive to analytical interferences such as heterophile antibodies, human anti-animal antibodies (HAMA), anti-analyte autoantibodies, matrix effect, hook effect, biotin, etc., which can cause false-positive or false-negative results [248]. Difficulties in interpreting data may also be due to frequent variability between methods. The lack of standardization and harmonization causes differences among the results obtained from the same samples by different IMAs, as well as discrepancies in reference intervals and cut-offs [249]. The scientific community, regulatory agencies, manufacturers and clinical laboratories should work together in order to minimize manufacturer-specific differences and optimize analytical performance [250]. To overcome these issues, in the last decades, other molecules have been studied such as Cyfra 21.1 [47]. Although some studies have shown their potential clinical application in ATC, overall they are not as useful as the well-known biomarkers widely used in routine [31]. However, current circulating tumor biomarkers for thyroid cancer are not always able to distinguish between benign and malignant neoplasms or between low-risk and high-risk malignant lesions in the preoperative phase [17]. In addition, they are not specific since they can be high even in non-neoplastic conditions [249].

In this context, the post-analytical phase, which concerns the reporting and the interpretation of the result, is fundamental. Indeed, the choice of the appropriate reference interval and/or the clinical decision limit (cut-off) of the biomarker is crucial [251]. The reference interval is a statistical calculation based on the determination of 2.5 and 97.5 percentiles obtained in a sample of unaffected subjects selected from the general population for a given biomarker [252]. The reference interval is defined by laboratory experts according to a well-defined consensus [251]. The clinical cut-off is quite different. It addresses a decision about a clinical condition in a precise clinical patient group. The clinical cut-off derives from clinical outcome studies, guidelines and consensus values, predictive values and ROC (receiver-operating characteristic) curves [251]. The optimal cut-off value for detecting cancer with 100% sensitivity and 100% specificity does not exist because for many cancer biomarkers, the values of the two groups of individuals often overlap [253]. This is even more difficult if cancer patients need to be distinguished from the group of patients with organ-related non-malignant diseases [253]. Therefore, the identification of a specific cut-off for thyroid tumor biomarkers is essential for the correct diagnosis of patients.

Another important aspect is the improvement of the technology that has allowed the development of genomic, transcriptomic and proteomic assays enabling the identification of a multitude of biomarkers which reflect a high signature of the molecular profile for each type of tumor [254]. The general aim is to identify novel circulating biomarkers that can supplement information from the currently used biomarkers. Regarding this aspect, CTCs and EVs-derived and free ctNAs offer a great possibility to assess genotypic features of cancer, besides observing cancer progression and treatment response, without the need for invasive biopsy thanks to their presence in the peripheral blood. Indeed, the evidence of the existence of ctDNAs and ctRNAs and the dysregulation of ccf-mtDNA, mRNAs, miRNAs, lncRNAs and circRNAs as well as proteins in several types of thyroid neoplasms offer the possibility of their potential use in clinical practice. The combination of high-throughput assays aimed at identifying the common mutations such as BRAF, RET, and RAS and at evaluating ctNAs can provide important information about the mutational spectrum of the tumor and guide the selection of appropriate targeted therapy. Indeed, the development of molecular technologies and NGS techniques allows us to characterize blood ctNA markers more deeply for the diagnosis, monitoring and prognosis of cancer [160]. Here, we have listed plenty of potential biomarkers sorted into different types of thyroid tumors. However, the literature data show limitations in their possible practical use. Firstly, most of them are general cancer markers. For instance, among miRNAs, miR-375 and miR-144, miR-221, miR-222, and miR-21 are found to be dysregulated in glioblastomas, breast, prostate, lung, colorectal, stomach, pancreatic, hepatic and neuroendocrine tumors [175,255,256,257]. In addition, the data currently available in the literature are derived from experimental studies or small-scale clinical trials in specific patient cohorts. Therefore, large-scale studies are required to confirm and validate their use in clinical practice. Furthermore, the current clinical use of CTCs and ctNAs is limited and still requires a complete integration of ctNAs as tumor-derived liquid biopsy markers. Thereby, several studies are needed to carefully identify circulating biomarkers and to design a panel of dysregulated ctNAs specific for thyroid cancer able to discriminate between malignant and benign nodules and, in case, between the different types of thyroid cancer. Moreover, combining several kinds of circulating markers (e.g., the well-known thyroid tumor proteins, CTCs, and ctNAs) in a single diagnostic panel may probably be helpful. Thus, the current challenge aims to improve the current expertise in using ctNAs as blood clinical markers to screen for thyroid neoplasms and detect minimal residual disease.

## 6. Conclusions and Future Perspectives

Recent findings in the field of tumors are paving the way for a new era of personalized and precision medicine by improving prognosis and quality of life for individuals. The discovery of new biomarkers of thyroid cancer can contribute to early disease detection, careful follow-up and personalized treatments by maximizing therapeutic results [31]. Furthermore, early diagnosis of thyroid cancer allows for less invasive surgery with consequent lower risks for the patient and may also avoid RAI ablation. Moreover, the ability to distinguish between benign and malignant lesions could help in choosing the most appropriate approach. Tailored to the specific needs of each patient and type of cancer, a targeted therapeutic regimen requires medical supervision to prevent adverse effects and maintain maximum effectiveness and quality of life. In thyroid cancer, personalized treatment can interfere with the development and multiplication of cancer cells by using specialized means to identify the molecules involved in the growth of cancer [258]. Tyrosine kinase inhibitors, immunotherapy and gene-targeted therapy are some of the main targeted treatments for thyroid cancer [31] that currently play a supporting role to the well-established surgical therapy and RAI ablation in advanced or treatment-refractory thyroid tumors [259]. Despite progress in recent years, much remains to be explored in the field of cancer and in particular in thyroid cancer to overcome current obstacles to the clinical application of new biomarkers [260]. In particular, future research should focus on the following areas: (1) development of standardized protocols for the validation of biomarkers to demonstrate reliability and reproducibility of assays in different conditions [261]; (2) support for collaborative multi-centre research initiatives that allow for the expansion of study sample numbers, resulting in faster and more convincing data on thyroid cancer [31]; (3) integration of genomic, transcriptomic, proteomic and metabolomic data to increase the sensitivity and specificity of biomarkers [3]; (4) advancement of bioinformatics tools and machine learning algorithms to better understand new biomarker models [262]; and (5) study of potential combinations of biomarkers with consolidated therapeutic strategies to ensure treatment efficacy and to predict treatment response [260].

## Figures and Tables

**Table 1:** List of well-known circulating biomarkers for the management of thyroid tumor disease. TG, thyroglobulin; CT, calcitonin; CEA, carcinoembryonic antigen; Ca 19-9, cancer antigen 19-9; DTC, differentiated thyroid carcinoma; MTC, medullary thyroid carcinoma; IMAs, immunoassays; LC-MS/MS, liquid chromatography coupled to tandem mass spectrometry.

Biomarker	Thyroid Tumor	Biological Matrix	Currently Available Methods	Clinical Use	Limitations	References
TG	DTC	Plasma, Serum	IMAs, LC-MS/MS	Prediction of tumor relapses after treatment (surgery, RAI ablation); estimation of tumor burden.	Elevations in non-neoplastic disorders; analytical interferences.	[28,47,48,49,50,51,52,54,64,65,66,67,68,69,70]
CT	MTC	Plasma, Serum	IMAs	Preoperative MTC identification (most sensitive marker); estimation of tumor burden; prognostic predictor; evaluation of response to therapy.	Elevations in non-neoplastic disorders; lack of a univocal cut-off for basal CT; analytical interferences.	[35,74,76,77,78,80,81,83]
CEA	MTC	Plasma, Serum	IMAs	Prognostic predictor; marker of tumor dedifferentiation, progression and invasion; evaluation of response to therapy.	Elevations in non-neoplastic disorders; lack of specificity for thyroid cancer (other neoplasms such as colon, breast, …); analytical interferences.	[89,90,91,92,93,94,95,97,98]
Ca 19-9	DTC, MTC	Plasma, Serum	IMAs	Prognostic predictor; marker of MTC dedifferentiation and disease aggressiveness.	Elevations in non-neoplastic disorders; lack of specificity for thyroid cancer (other neoplasms such as pancreas); lack of a univocal cut-off to distinguish between benign and malignant disease; analytical interferences.	[107,108,109,110,111,112,113,114,115,116,121]

## Data Availability

No new data were created or analyzed in this study. Data sharing is not applicable to this article.

## Abbreviations

The following abbreviations are used in this manuscript:

ATC	Anaplastic thyroid carcinoma
ATG	Anti-thyroglobulin antibodies
ATPO	Antibodies against thyroid peroxidase
BMI	Body mass index
BST2	Bone marrow stromal cell antigen 2
Ca 19-9	Carbohydrate antigen 19-9
CCH	C-cell hyperplasia
ccf-mtDNA	Cell-free mtDNA
CEA	Carcinoembryonic antigen
circRNAs	Circular RNAs
CLEIA	Chemiluminescence enzyme immunoassay
CLIA	Chemiluminescence assays
CT	Calcitonin
CT-CALCA	CT-related polypeptide alpha transcript
CTCs	Circulating tumor cells
ctDNAs	Circulating tumor DNAs
ctNAs	Circulating tumor nucleic acids
ctRNAs	Circulating tumor RNAs
CV	Coefficients of variation
DTC	Differentiated thyroid carcinoma
ECLIA	Electrochemiluminescence assays
ELISA	Enzyme-linked immunosorbent assay
ELFA	Enzyme-linked fluorescence assay
EQA	External quality assessment
EVs	Extracellular vesicles
FIA	Fluoroimmunoassay
FEIA	Fluorescence enzyme immunoassay
FMTC	Familial medullary thyroid cancer
FNAB	Fine needle aspiration biopsy
FTC	Follicular thyroid carcinoma
GFRA2	Glial cell line-derived neurotrophic factor family receptor alpha-2
HAMA	Human anti-animal antibodies
hsTG	TG assays with high analytical sensitivity
IYD	Iodotyrosine deiodinase
ILMA	Immunoluminometric assay
IMAs	Immunoassays
IRMA	Immunoradiometric assay
IRP	International Reference Preparation
LC-MS/MS	Liquid chromatography coupled to tandem mass spectrometry
lncRNAs	Long non-coding RNAs
LNM	Lymph node metastasis
LOD	Limit of detection
MEN	Multiple endocrine neoplasia
mRNAs	Messenger RNAs
miRNAs	MicroRNAs
MTC	Medullary thyroid carcinoma
mtDNA	Mitochondrial DNA
ncRNAs	Non-coding RNAs
NENs	Neuroendocrine neoplasms
NGS	Next-generation sequencing
OTC	Oncocytic thyroid carcinoma
PPIs	Proton pump inhibitors
PPV	Positive predictive value
ProCT	Procalcitonin
PTC	Papillary thyroid carcinoma
PTMC	Papillary microcarcinoma
RAI	Radioactive iodine
rhTSH	Recombinant human TSH
RIA	Radio immunoassay
ROC	Receiver-operating characteristic
TG	Thyroglobulin
T3	Triiodothyronine
T4	Thyroxine
TPO	Thyroid peroxidase
TRACE	Time Resolved Amplified Cryptate Emission
TSH	Thyrotropin
TSHR	Thyroid-stimulating hormone receptor
